# Effective Management of Ketamine-Induced Bladder Syndrome With Baclofen During Ketamine Detoxification: A Case Report

**DOI:** 10.1192/bjo.2025.10724

**Published:** 2025-06-20

**Authors:** Foroughozzahra Esmaeilpour, Mounika Iderapalli, Edwin Ugoh

**Affiliations:** Essex Partnership University Trust, Chelmsford, United Kingdom

## Abstract

**Aims:** Ketamine, a dissociative anaesthetic, is used therapeutically to treat mental health disorders like depression, anxiety, and PTSD. However, its recreational misuse can lead to severe physical and psychological consequences. A concerning long-term effect of ketamine misuse is ketamine-induced bladder syndrome (KIBS), which presents with symptoms such as urinary urgency, frequency, and pain. As dependence on ketamine develops, the severity of KIBS increases, potentially leading to significant organ damage. In such cases, ketamine detoxification becomes essential for reducing or eliminating ketamine from the system while managing withdrawal symptoms and psychological distress. While no FDA-approved medication exists for ketamine detoxification, treatment generally focuses on addressing withdrawal symptoms, psychological and physical dependence, and related complications.

**Methods:** This case involves a 23-year-old woman with a complex mental health history, including ADHD, Borderline Personality Disorder (BPD), anxiety, and previous self-harm attempts, well-documented by community mental health services. Her substance use began at age 18, starting with ketamine use during university. Over time, her ketamine consumption escalated from 0.5 grams every few months to 3.5–5 grams, causing both physical and psychological harm. Her usage pattern varied; sometimes she refrained from use for a day or two, but often she would use it continuously for 3 to 4 days, followed by a break. At times, she alternated days of use, with usage depending on her mood, occasionally taking the entire dose at once.

Approximately four years after initiating ketamine use, she developed significant bladder pain, frequent urinary tract infections (UTIs), and a constant urge to urinate. To manage these symptoms, she began using pregabalin, initially obtained illegally but later prescribed at a dosage of up to 600 mg daily by her GP. Despite this, her bladder pain remained inadequately controlled, and she resorted to using paracetamol and co-codamol, which proved ineffective. She also visited a urologist biweekly. She underwent two private ketamine detoxifications in 2021, although relapse occurred a few days later.

Considering her ketamine addiction and bladder pain, she underwent detoxification treatment. Managing her bladder pain was a key focus of her treatment. Diazepam was prescribed for anxiety, and in addition to pregabalin, baclofen was added to address the bladder pain. During detox, the patient reported significant improvement in bladder pain.

**Results:** One of the most challenging aspects of ketamine-induced bladder syndrome is the chronic bladder pain. Baclofen, a GABA-B receptor agonist, helped relax smooth muscles, including those in the bladder, reducing spasticity and discomfort. Baclofen also alleviated neuropathic pain, which contributed to ketamine-induced bladder dysfunction. Baclofen’s ability to manage neuropathic pain makes it a valuable option when traditional painkillers fail. By inhibiting pain signal transmission, baclofen provides relief from discomfort and reduces urinary urgency and frequency. In this case, baclofen effectively alleviated bladder pain, reducing reliance on pregabalin during detoxification. Despite the complexities of her treatment plan, baclofen was vital in improving the patient’s quality of life during detox.

**Conclusion:** Baclofen proves to be an effective alternative for managing ketamine-induced bladder pain, particularly when traditional treatments fail. This case emphasizes the importance of a multidisciplinary approach to address both physical and psychological challenges during ketamine detoxification. By managing bladder pain and other withdrawal symptoms, patients can experience a sustainable detox process, ultimately improving their chances of recovery and decreasing the risk of relapse.

